# The Role of Internet Information on Anti-HPV Vaccines: A Comprehensive Overview of a Double-Edged Sword

**DOI:** 10.3390/vaccines13050445

**Published:** 2025-04-23

**Authors:** Luca Giannella, Camilla Grelloni, Leonardo Natalini, Gianmarco Sartini, Federica Lavezzo, Camilla Cicoli, Marco Bernardi, Mila Bordini, Martina Petrini, Jessica Petrucci, Tomas Terenzi, Giovanni Delli Carpini, Jacopo Di Giuseppe, Andrea Ciavattini

**Affiliations:** Woman’s Health Sciences Department, Gynecologic Section, Polytechnic University of Marche, 60123 Ancona, Italy; luca.giannella@ospedaliriuniti.marche.it (L.G.); c.grelloni@pm.univpm.it (C.G.); l.natalini@pm.univpm.it (L.N.); g.sartini@pm.univpm.it (G.S.); f.lavezzo@pm.univpm.it (F.L.); camilla.cicoli@pm.univpm.it (C.C.); m.bernardi@pm.univpm.it (M.B.); m.bordini@pm.univpm.it (M.B.); martina.petrini@pm.univpm.it (M.P.); j.petrucci@pm.univpm.it (J.P.); t.terenzi@pm.univpm.it (T.T.); giovanni.dellicarpini@ospedaliriuniti.marche.it (G.D.C.); jacopo.digiuseppe@ospedaliriuniti.marche.it (J.D.G.)

**Keywords:** cervical cancer, HPV vaccine, internet, social media, misinformation

## Abstract

Cervical cancer (CC) is the only cancer that has the possibility of primary and secondary prevention. Despite this, it is one of the leading causes of cancer death among women, especially in developing countries. The World Health Organization has set the ambitious goal of eliminating CC by 2030 by suggesting specific types of intervention. Unfortunately, to date, we are very far from this goal at a global level, including developed countries. Implementing vaccination coverage among the target population is one of the strategies to be pursued in this area. Achieving this goal should include combating misinformation about the HPV vaccine, which is one of the main reasons for vaccination hesitancy. Such conspiracy theories are prevalent on social media, one of the primary sources of information for adults and adolescents today. In this regard, the Internet plays a significant role in disseminating information about the HPV vaccine, both positively and negatively. The Internet provides easy access to information about the HPV vaccine, including its safety, efficacy, recommended dosing schedule, and potential side effects. It may promote vaccine advocacy and debunking vaccine myths. On the other hand, the Internet may be the place for disseminating misinformation and influencing vaccine decision making. It is a double-edged sword in shaping public discourse and perceptions about the HPV vaccine. This overview aims to assess the literature on this topic in depth to promote evidence-based information, analyze the social channels through which misinformation spreads, and leverage digital health interventions essential for promoting HPV vaccination and reducing the burden of HPV-related diseases.

## 1. Introduction

Cervical cancer (CC) is the only oncological disease that has primary prevention. The development of HPV vaccines has been a game changer in this regard. The bivalent vaccine, licensed by the FDA in 2006, contains HPV genotypes 16 and 18 [1]. This was followed by the introduction of the quadrivalent and nonavalent vaccines in 2007 and 2014, respectively, covering HPV genotypes 6, 11, 16, 18, 31, 33, 45, 52, and 58 [1,2]. The nonavalent vaccine, targeting seven high-risk HPV genotypes, has the potential to prevent a staggering 90% of CC cases, offering a beacon of hope in the fight against this disease [3,4]. Despite this promising outlook, CC remains a significant health concern, particularly in developing countries [5].

In August 2020, the World Health Organization (WHO), a global leader in public health, set the ambitious goal of eliminating cervical cancer (less than 4 cases per 100,000 women per year) by 2030 [6]. This objective underscores the urgency of the situation. The WHO defined the cut-offs for primary and secondary prevention for the target population as follows: 90% vaccination coverage and 70% adherence to screening [6]. However, the stark reality is that global data clearly show we are far from this goal, with significant disparities in CC rates between developed and developing countries [7]. At the end of 2022, to increase vaccination coverage at lower costs, the WHO recommended administering a single dose of vaccine as effective for preventing CC [8].

To achieve this goal, in addition to promoting the benefits and safety of the HPV vaccine, it is necessary to counter the specific misinformation circulating about it, such as unfounded claims about its safety or effectiveness, which represents one of the main obstacles to vaccination coverage [9].

Nowadays, social media are a tool for disseminating information, even in the scientific field. These digital environments foster interaction and the formation of communities based on shared interests, strengthening the sense of security and trust in vaccines and their usefulness. Indeed, group discussion can increase vaccine trust, consolidating learned information and dispelling uncertainties [10,11]. These means of communication are often used as the preferred research method for teenagers, as they allow the problem to be researched directly, easily, and anonymously, avoiding the embarrassment of confrontation [12,13].

Social media promote vaccination through personal testimonials or stories with higher engagement than informative posts [11]. One of the most striking historical examples was the public announcement of HIV positivity by NBA star Earvin “Magic” Johnson in 1991. Epidemiological studies have shown that this event catalyzed public awareness of the risk of infection, and there have since been significantly higher rates of condom use and HIV testing, particularly among black and Hispanic populations, increasing the rate of early diagnosis and improving survival rates by about 10 years for each new patient [14,15].

However, there is also another side of the coin to be highlighted. Social media are fertile ground for the birth and spread of conspiracy theories and false scientific beliefs, which mainly reach adults, parents, and adolescents and impact vaccination coverage [9,16].

The dissemination of misinformation about the HPV vaccine via the Internet and social media is a significant public health challenge [17]. Wilson and Wiysonge’s research demonstrated that social media are frequently used to organize anti-vaccination campaigns, leading to increased mistaken beliefs about vaccine safety and, in some cases, a reduction in vaccination rates by up to 2% per year [9]. This evidence highlights the need for strategies promoting evidence-based information to counteract vaccine hesitancy, providing a solid and reliable approach to addressing this issue.

Previous reviews showed negative messages often concern side effects, vaccine safety, and poor efficacy [12,18,19,20]. In turn, this message was associated with a higher frequency of vaccination refusal, decreased vaccination coverage, and a greater sharing of these critical issues with other users online [12,18,19,20]. In contrast, positive messages often concern the importance of the vaccine in preventing and protecting from pre-invasive and invasive diseases of the lower genital tract. Rarely, positive messages focus on the safety and efficacy of the vaccine [12,18,19,20]. It is worth noting that pro-vaccination messages are associated with higher HPV vaccination coverage. Given the real-world impact, several authors consider this topic understudied [12,18,19,20]. It could represent an effective tool for expanding knowledge about HPV and HPV vaccination by trying to reach parents and adolescents appropriately. In this last area, research is considered lacking.

This overview aims to evaluate the literature on this topic, analyze the social channels through which misinformation circulates, promote evidence-based information, and implement digital health policy interventions to promote HPV vaccination and reduce the burden of HPV-related diseases.

## 2. Methods

Our research adhered to the SANRA (Scale for the Assessment of Narrative Review Articles) guidelines, ensuring the quality and reliability of our findings. We conducted a comprehensive literature search using the Pubmed, Scopus, and Web of Science databases [21]. The search was focused on the following terms: (Human Papillomavirus Vaccine) AND (Internet OR social media) AND (misinformation OR disinformation) in Pubmed (all fields) (accessed on 15 February 2025) and Scopus and Web of Science (Title/Abstract/Keywords) (accessed on 15 February 2025) databases. The research time frame spanned from January 2006 (the release of the first HPV vaccine) to December 2024, with the only filter being English. We included relevant papers—original and review articles— providing data strictly related to the HPV vaccine and misinformation on the Internet/social media. Studies including other factors for or against HPV vaccination not related to HPV vaccine misinformation data from online information were excluded (e.g., articles including HPV vaccine acceptance or hesitancy in newspapers, in magazines, on TV, in interviews, and in surveys), along with case reports and articles in non-English languages. The papers included in the qualitative analysis were obtained in full-text format and screened for additional references.

The main outcomes and topics of our research were the following: (i) a comprehensive overview of HPV vaccine misinformation on the Internet/social media in the considered time frame; (ii) the impact on parents and adolescents (target population) regarding the choice to get vaccinated based on misinformation about the HPV vaccine; and (iii) provide strategies to combat misinformation.

The study selection process was carried out to ensure the highest quality of research. Four independent reviewers (Camilla Grelloni, Giammarco Sartini, Federica Lavezzo, and Luca Giannella) employed a 2-step screening method. Initially, titles and abstracts were scrutinized to assess for eligibility using the inclusion criteria, excluding irrelevant studies. Subsequently, the four reviewers thoroughly evaluated the full texts of the included articles to assess for study eligibility using the inclusion criteria and avoid duplications of the included cases. To further enhance the comprehensiveness of this study, four other authors (Jacopo Di Giuseppe, Giovanni Delli Carpini, Camilla Cicoli, and Leonardo Natalini) manually searched reference lists to search for additional relevant publications. Other authors (Marco Bernardi, Mila Bordini, Martina Petrini, Jessica Petrucci, and Tomas Terenzi) contacted the authors of papers unavailable online based on our sources for the full text.

Data collection was study-related (author(s) and year of study publication) with the following items (article type, author nationality, country of data origin, information topics associated with vaccine hesitancy, information topics not associated with vaccine hesitancy, critical issues faced by health national or international agencies on online information).

A descriptive analysis of the data was performed. Furthermore, the correlation between the year of publication and number of articles was evaluated in the considered time interval. Continuous variables were tested for normal or non-normal distribution using the Kolmogorov–Smirnov test. When the distribution of variables was not normal, the degree of relationship between the variables was determined using rank correlation (Spearman’s coefficient). MedCalc^®^ Statistical Software version 20.305 (MedCalc Software Ltd., Ostend, Belgium, 2023; https://www.medcalc.org) was used.

**Figure 1:**
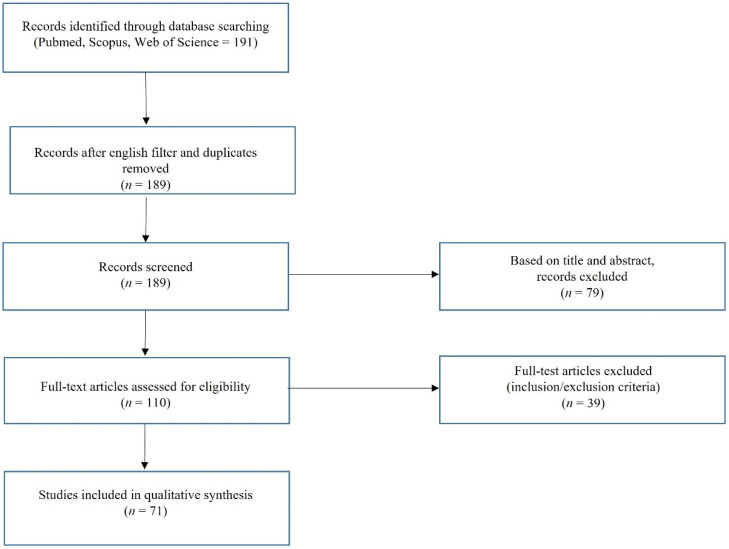
Literature review flowchart.

Figure 1 shows a flowchart of the literature review process. The Supplementary Material provides a detailed summary of the selected studies (Table S1) [12,18,19,20,22,23,24,25,26,27,28,29,30,31,32,33,34,35,36,37,38,39,40,41,42,43,44,45,46,47,48,49,50,51,52,53,54,55,56,57,58,59,60,61,62,63,64,65,66,67,68,69,70,71,72,73,74,75,76,77,78,79,80,81,82,83,84,85,86,87,88].

## 3. Internet Misinformation on HPV Vaccine

In the initial stages of the HPV vaccination campaign, one of the primary media challenges was erroneous assumptions, propagated mainly by some ethnic–religious groups, that the vaccine would lead to a substantial increase in sexual promiscuity among young people, in terms of the number of partners and the number of episodes [89]. Conversely, Kudo R et al. [90], in their Internet survey administered to 828 young Japanese women aged from 16 to 20 years, found that unvaccinated women exhibited riskier sexual behaviors, linking this result to the poor knowledge of the risks associated with HPV infection within this population.

Among other widespread false beliefs about the HPV vaccine, which a substantial body of research has refuted, are reduced fertility rates in vaccinated individuals, increases in cases of childhood developmental disorders, the presence of toxic ingredients or nanobots in the administered vaccines, and even an elevated risk of death [47,89].

One of the most widely applied theories in healthcare contexts, the Health Belief Model (HBM), succinctly encapsulates these concerns [11,13]. It comprises four constructs:

- Knowledge and perception of the severity of the disease and one’s vulnerability to it;
- Perception of the benefits and obstacles related to vaccination;
- Self-efficacy or confidence in one’s ability to get vaccinated;
- External signals that prompt action.

The absence of these factors indicates a tendency towards delayed or refused vaccination. These virtual communities exploit the ‘long-tail’ effect, as defined by Wilson et al. [9]. The ‘long-tail’ effect refers to the lack of cultural selection filters on the Internet, allowing fringe groups with niche interests, such as the anti-vaccine movement, to disseminate their ideas to a broader audience. In other words, the ‘long-tail’ effect enables the proliferation of anti-vaccine narratives by providing a platform for these fringe groups to reach a wider audience than they would in traditional media. This effect has significantly contributed to the spread of anti-vaccine narratives.

In their qualitative multi-method research design on the HPV vaccine, Boatman et al. [88] analyzed 1.000 social media comments present on Facebook (*n* = 999), X (formerly Twitter) (*n* = 998), and TikTok (*n* = 999). The proportion of misinformation comments identified on these three platforms was 41.1%, 28.6%, and 9.6%, respectively. Of the negative comments, 27% were observed under the posts of the most active initial creators. In particular, the authors highlighted that on Facebook and X, the primary focus of discussion was conspiracy theories, in contrast to TikTok, where the prevailing topic was the vaccine’s adverse effects.

Additionally, Ekram et al. [87] identified a high prevalence of anti-vaccine videos on YouTube (57%) compared to pro-vaccine videos (31%) and neutral-tone videos (11%). However, they noted that the video’s tone did not significantly predict its popularity, as measured by the number of likes, dislikes, views, and comments.

The impact of exposure to anti-vaccine materials on vaccination rates is a matter of grave concern. Ortiz RR et al. [12] observed a significant increase in vaccine refusal and a tendency to share harmful content on social media among individuals exposed to the anti-vaccine narrative. Dunn et al. [76] investigated on X the potential correlations between HPV vaccination coverage data in each American state, sociocultural factors, and exposure to anti-vaccine messages. The only factors that exhibit a strong correlation with HPV hesitancy were identified as low levels of education and exposure to anti-vaccine topics. A few years later, Calo WA et al. [56] confirmed the same results obtained on X.

**Figure 2:**
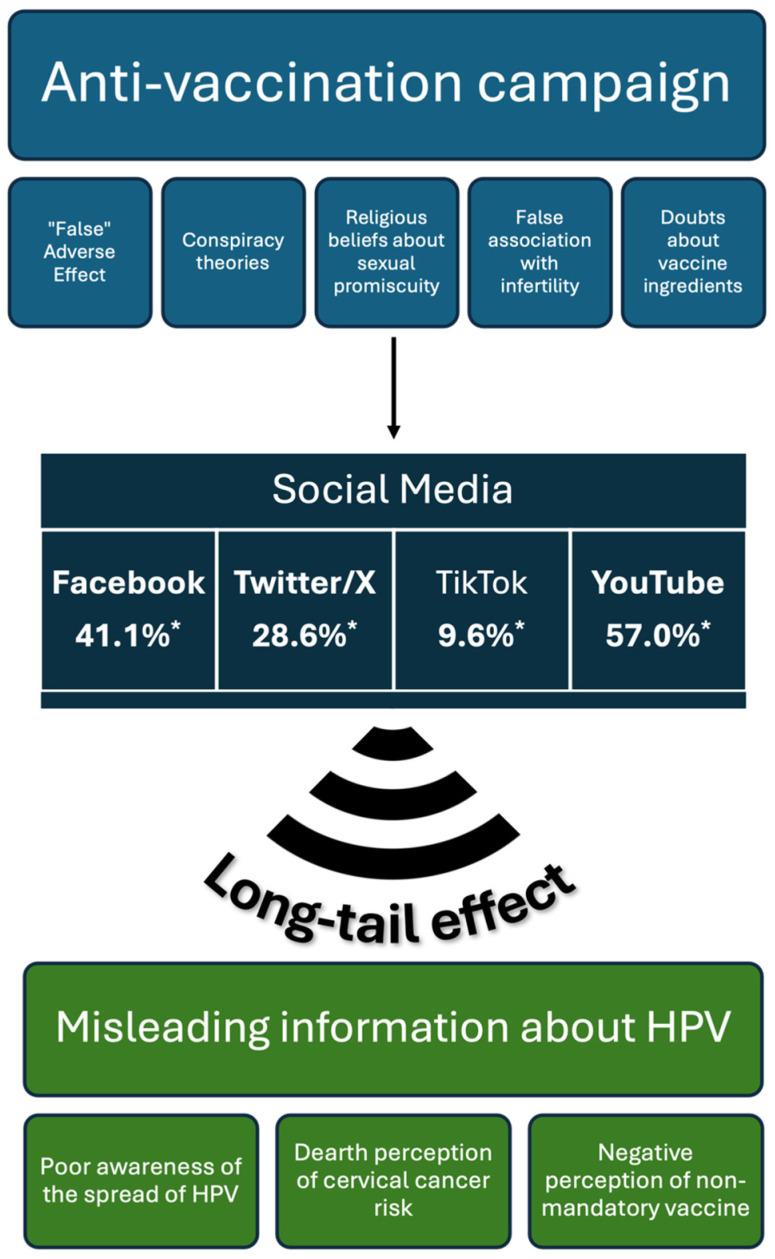
The main topics of the anti-HPV vaccine campaigns that, through the long-tail effect of social media, produce misleading information [87,91]. * = percentage of posts/comments/videos concerning misinformation on the total about HPV vaccination. Icons were obtained from www.Flaticons.com.

Figure 2 shows the main misinformation campaigns that lead to misleading beliefs about the HPV vaccine through social media.

**Figure 3:**
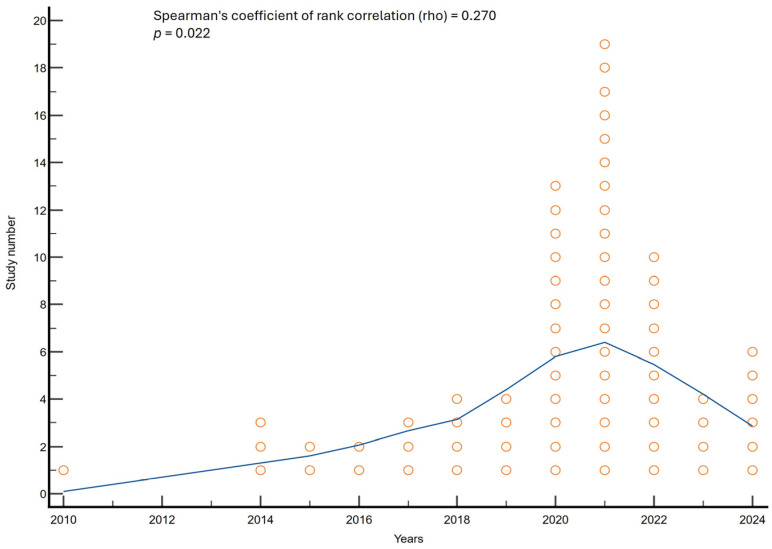
Correlation between the number of studies and year of publication.

The present research found additional information. There is a positive correlation between the number of studies on online misinformation about the HPV vaccine and the year of their publication, starting from its release (Figure 3). Figure 3 shows researchers’ peak interest in 2020–2022, likely driven by the COVID-19 pandemic. Subsequently, a decrease in published articles can be noted. Post-pandemic data can be confusing to interpret. A decrease in articles published on the matter was to be expected immediately after an extraordinary event in which misinformation was the main topic. However, monitoring the interest in this question in the coming years is of great importance since, as mentioned above, this topic needs to be explored further, especially in monitoring and providing effective strategies against misinformation.

Furthermore, based on our data (Table S1), the main misinformation topics associated with vaccine hesitancy include conspiracy theories, side effects, mistrust of authority, and misleading interpretation of the information source. On the contrary, among the information topics not associated with vaccine hesitancy, the most frequent are represented by pro-vaccination messages from authoritative sources, positive personal stories, fear about the consequences of HPV infection (disease, treatments), and instruction/cultural factors. From these data, it appears evident that the individuals most likely to reject misinformation are those who have a positive or neutral attitude towards the topic, usually of high educational level, and therefore trust messages coming from authoritative sources and from positive personal stories and fear the consequences of a possible illness. Recovering users who accept conspiracy theories is challenging regarding information associated with vaccine hesitancy. On the contrary, refusing vaccination due to fear of side effects, mistrust of authorities, or scientific misinformation are topics on which action can be taken to reduce vaccine hesitancy. Therefore, more than misinformation itself, attitudes, personal beliefs, and sociocultural factors determine the impact of misleading online messages on the real world. However, these data may be of significant importance, especially for national and international health agencies, as they represent priority issues to focus on to reduce vaccine refusal. Regarding these health agencies, 8 of the 71 studies included in this review found critical issues regarding their role in poor monitoring of online disinformation and their lack of responses to comments or questions. These critical issues can increase vaccine hesitancy in parents and adolescents, who may interpret them as lacking information and unclear transparency.

**Figure 4:**
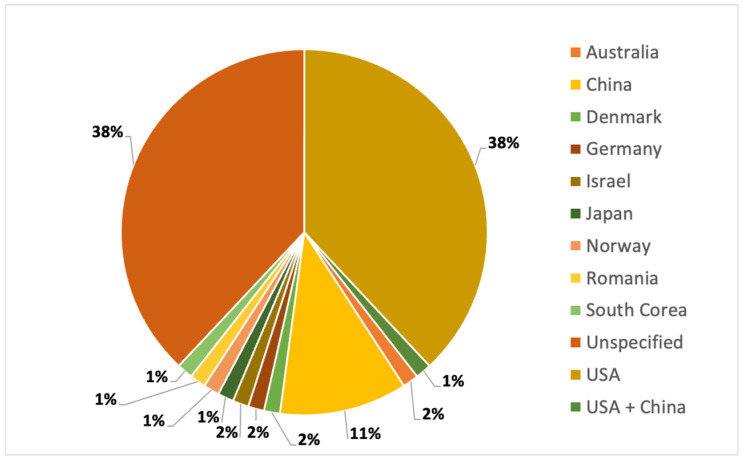
Percentage of country of data origin.

Regarding the country of data origin, most are unspecified (39%, probably reflecting an unrestricted search from all over the world), followed by the USA (38%) and China (11%) (Figure 4). The only two comparable countries are the USA and China, and there are no significant differences in information topics that favor or do not favor vaccine hesitancy. However, it is interesting to note that about 90% of the Chinese data focus only on topics against misinformation (Table S1). There may be multiple explanations for this difference compared to other countries. Omitting misinformation can lead to less publicity for negative messages, thus reducing their possible further spread. Another explanation relates to different governance methods, where what is generally considered misinformation has a more limited space in some countries than others. This last aspect underlines how the use and messages of social media can differ significantly depending on the country.

In summary, the type of information spread on social media may depend on the country of data origin. Furthermore, knowing whether online information is associated with vaccine hesitancy can represent priorities to work on, given that misinformation contributes to a reduction in vaccination rates, hindering HPV prevention. This phenomenon is intertwined with the broader influence that the Internet exerts on people’s opinions, particularly on adolescents and their parents.

## 4. Influence of Social Media on Adolescents and Parents Regarding HPV Vaccines

### 4.1. Influence on Parents

The impact of social media on parents regarding HPV vaccination of their children is significant. YouTube is the most used platform by parents in the United States (88%), followed by Facebook (79%) and finally Instagram (47%) [92]. In 2022, approximately 60% of adults in the United States (152.3 million) viewed health information videos through online platforms, leading to an increase from 2017 to 2022 of approximately 100%. These users were mainly adults between 18 and 40 years of age with a high level of education [93].

A study by Lama Y et al. in 2021 found that parents who engaged in various social media behaviors, such as networking, sharing information, participating in health-related forums, and watching health-related videos on YouTube, had a higher level of awareness about HPV vaccination [94].

There is a correlation between the type of information acquired on social media about HPV vaccination and how this can influence parents’ decisions to vaccinate their children [95]. Regarding the topic, Llavona-Ortiz et al. published the results of a web-based survey about the possible effects of social media on parents regarding vaccine decisions for their adolescent children [31]. They observed that parents who acquired positive information regarding HPV vaccination were likelier to have started an HPV vaccination program for their children (OR 1.74, 95% CI 1.24–2.44). Parents exposed to information against HPV vaccine were more likely to delay (OR 3.29, 95% CI 1.66–6.51) or not perform (OR 4.72, 95% CI 2.35–9.50) vaccination for their children. Additionally, misleading information about the benefits of HPV vaccination has proven to be a significant factor in delaying starting vaccination in adolescent children [31].

A study by Gilkey et al. observed that 28% of parents refused vaccination, 8% delayed it, and 64% took a neutral position, neither delaying nor refusing vaccination [73]. The authors noted that the possibility of refusing vaccination was lower among parents who had understood the efficacy of the HPV vaccine. On the contrary, the refusal to vaccinate their children was higher among parents who had doubts about the safety of the vaccine, perceiving vaccination as a potential harm. Finally, parents who had developed an opinion of uncertainty about the HPV vaccine showed a more significant delay in carrying it out (RRR 1.76, 95% CI 1.08–2.85) [96].

**Figure 5:**
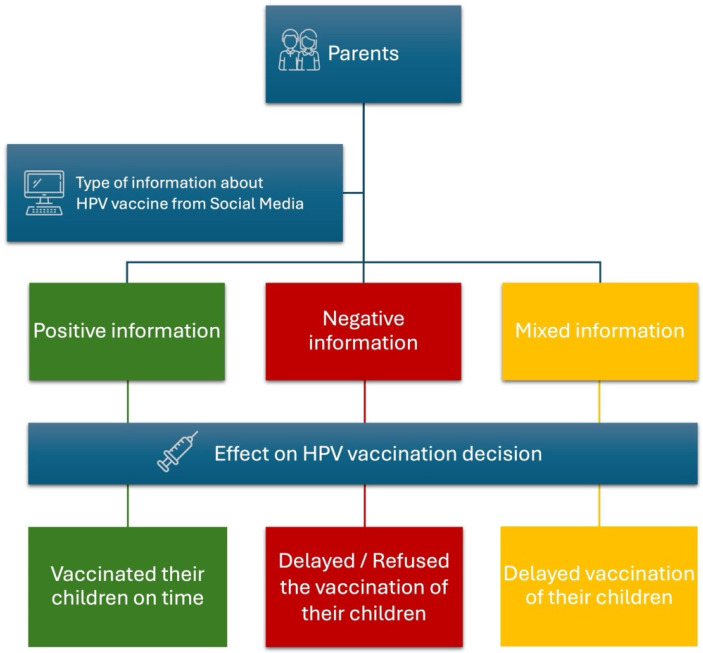
Impact of HPV vaccine information spread on social media and parents’ vaccination decisions for their children [31]. Icons were obtained from www.Flaticons.com.

In their online survey, Margolis et al. found that stories about vaccination-related harm come more often from social and traditional media, and stories of preventable disease come more often from conversations. Moreover, coming across stories of disease prevention was only sometimes associated with greater adherence to the vaccination program [73]. Confirming the data previously illustrated, parents who heard only stories about the adverse effects of the HPV vaccine were less likely to have initiated HPV vaccination for their adolescent children (aOR 0.48, 95% CI 0.33–0.69) and were more likely to have delayed (aOR 2.00. 95% CI 1.09–3.71) or refused (aOR 8.87, 95% CI 4.09–19.25) HPV vaccination [73]. Figure 5 summarizes the effect of online information on parents’ decisions to vaccinate their children.

From the above, it is clear that social media significantly influence users’ behavior, even in the healthcare sector. This influence can be empowering, as parents and adolescents may choose to get vaccinated based on the information they acquire on social media, feeling confident and in control of their decisions.

### 4.2. Influence on Adolescents

Despite the growing body of evidence indicating that adolescents may be the primary decision makers regarding HPV vaccination, there is a paucity of studies in the literature that directly investigate their knowledge, awareness, or opinion about the topic. This underscores the urgent need for more research, emphasizing the importance of understanding adolescents’ perspectives on HPV vaccination. This research is a matter of academic interest and a pressing need in public health [97].

A recent study conducted by the Pew Research Center surveyed 1453 American teenagers aged 13 to 17. The study aimed better to understand teenage populations’ social media habits and practices. The findings revealed that YouTube is the most popular online platform, with 93% of teenagers declaring they had used it at least once, followed by TikTok (63%), Snapchat (60%), and Instagram (59%). Compared to 10 years ago, there has been a significant decline in the use of Facebook and X (formerly known as Twitter). Specifically, the percentage of teenagers who used these platforms decreased from 71% in 2014–2015 to 33% in 2023 for Facebook and from 33% to 20% for X [98].

Facebook remains the most used online platform among parents of children between 9 and 14 years old, the age group recommended for HPV vaccination. Given that approximately half of teenagers use the Internet ‘almost constantly’, the potential influence of social media on adolescents’ perspectives on HPV vaccination cannot be overstated. It can significantly shape their attitudes, awareness, and behaviors [12].

Ortiz et al. recruited a group of adolescents and provided them with information regarding HPV and vaccination through social media, including Facebook. The results demonstrated that, compared to the control group, the intervention group displayed heightened interest and more profound knowledge and engaged in multiple discussions with their parents, peers, and healthcare professionals regarding HPV and vaccination [99].

In their systematic review and meta-analysis regarding factors associated with HPV vaccine uptake in teenage girls, Kessels et al. observed that girls who had been vaccinated had higher knowledge of HPV vaccination, HPV, and related cervical cancer compared to non-vaccinated girls [100].

In a cross-sectional, retrospective, observational study, Boatman et al. assessed the 170 most popular TikTok posts on HPV vaccination. Their findings revealed that the majority (53.5%) of TikTok posts expressed a pro-vaccination opinion, 16.4% expressed an anti-vaccination opinion, and 30.0% expressed no or a mixed opinion [91]. Regarding X, in an analysis of 3876 tweets, Kornides and colleagues identified that approximately a quarter (24%) of the content was against vaccination. This content predominantly addressed concerns related to potential side effects or perceived ineffectiveness of vaccines. On the other hand, the majority (76%) of the content expressed support for the HPV vaccine [34].

However, Kearney and colleagues analyzed 320 Instagram posts about HPV and HPV vaccination. They observed that 55.8% expressed negative opinions about vaccination, while 42.2% supported vaccination practice. It is noteworthy that, despite the greater quantity of posts in favor of HPV vaccination, the popularity of posts expressed by “number of likes” was higher for anti-vaccine content (24 vs. 86 likes; *p* < 0.001) [101].

**Figure 6:**
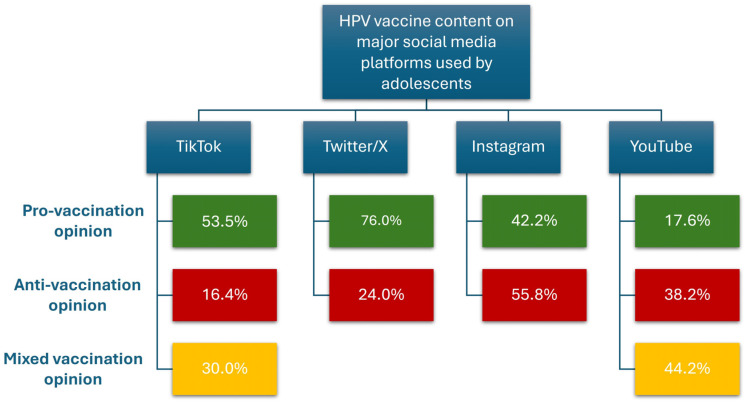
Pro-, anti-, and mixed HPV vaccination opinion rates on the most popular social media platforms used by teenagers [34,91,101,102].

Among YouTube visual content, 81.4% of videos provided general information related to HPV, discussing the relationship between HPV infection and cancer, and 64.3% addressed HPV screening. A total of 34 videos (49.3%) dealt with the topic of vaccination: 15 expressed a neutral opinion, while 6 encouraged and 13 discouraged HPV vaccination. Although few, this visual content was viewed 17 million times. These videos could potentially hinder efforts to increase HPV vaccination rates, which are already considerably below the target level [102]. Figure 6 summarizes the different opinions on the HPV vaccine spread by the leading social media platforms used by adolescents.

From the literature analyzed, it can be deduced that the mixed and not-always-clear information easily found on social media can significantly alter adolescents’ awareness about HPV vaccination and potentially influence their attitude towards starting a vaccination program.

## 5. Strategies to Combat Misinformation and Promote Evidence-Based Information

### 5.1. The Role of the WHO and Health Organizations on Social Media

Our literature review revealed that the social media content that most engages audiences is represented by positive personal narratives promoted by authoritative profiles with transparent scientific sources to support them.

In contrast, as shown by Coman et al., who explored conversations on X and Instagram regarding the HPV vaccine during HPV Awareness Day 2019, health organizations and their officials encouraged people to get the HPV vaccine. However, these messages did not contain narratives, and the posts received comments or questions that often went unanswered [28].

As the previous literature claims, web-based health communications cannot just deliver information unidirectionally, believing that audiences will accept the messages without doubt [39,103].

Zhang et al. demonstrated that it is not the content of HPV-promoting posts that emotionally engages the target audience but rather the comment section under those posts, in which users express both positive and negative sentiments, especially anger [39]. The most cited topics in comment sections concerned doubts about the safety and effectiveness of the vaccine and misinformation about the HPV vaccine. Users also asked further questions about the sources of information, suggesting that the target population actively wants to participate in the vaccine hesitation dialectic. However, public health profile pages did not provide answers directly in the comment section. In addition, a thorough examination of the comments written by the target population under these posts could be a valuable tool for understanding the erroneous beliefs that drive misinformation and producing relevant messages to address them [39].

Health organizations should also limit the circulation of HPV vaccine misbeliefs on social media platforms, which could influence vaccine hesitancy. This strategy should include active reporting by them on these platforms. Several social media platforms are trying to narrow the spread of HPV vaccine misinformation on their pages by implementing user standards for their sites (user policies or guidelines) [55]. Facebook, Instagram, Messenger, and Threads have a clear Community Standards policy about vaccines, and they combine users’ feedback and third-party fact checkers to detect potential health misbeliefs. At the same time, YouTube identifies “problematic content at scale” by mixing human feedback and machine learning [104,105].

Despite these efforts, the Center for Digital Hate calculated that 95% of posts against the HPV vaccine were not removed from social media platforms after the Center had reported these to the companies [106].

The WHO and other public health agencies should urge all social media sites to further tighten these Community Standards to prevent the spread of anti-vaccine content and speed up the process of monitoring, reporting, and removing misinformation posts.

Moreover, healthcare providers should be involved in screening mechanisms for posts containing false information about vaccines and should be provided with direct communication channels for reporting to social media companies.

In conclusion, the WHO and other government health agencies could play a key role in combating HPV vaccine misinformation on social media by strengthening the following:

- Communication methods: Use positive narratives and promote bidirectional dialectic in the comment section under posts;
- Surveillance methods: Implement more restrictive Community Standards on social media and more effective reporting mechanisms for misinformation posts.

### 5.2. Building a Tailoring Health Program for HPV Vaccination

Social media messages should consider the following factors: the messenger, the content, and the interpreter. Regarding the messenger, healthcare professionals could be more active in promoting and spreading positive messages on social media about the HPV vaccine. An organized and rigorous approach should be taken to analyze scientific information and correct misinformation through fact checking, providing reassurance and confidence in shared information [107]. The content of the message the healthcare professionals want to spread should use language adapted to the characteristics, concerns, and interests of their audiences, maintaining confidentiality and emphasizing that the HPV vaccine can prevent other types of cancer besides cervical cancer [108]. Healthcare organizations could create a network to share approaches, content, and outcomes. As previously shown in several studies, HPV vaccine narratives should be conveyed by multimodal messages composed of text, static ads, videos, podcasts, webinars, virtual reality conferences, and pro-and-con sessions to engage and persuade audiences [32,61,109,110,111]. In a qualitative study, multi-disciplinary school-based HPV health promotion programs consisting of educational videos, animations, digital games, and booklets generated confidence in vaccine safety and effectiveness. These boosted vaccination intention among students [112].

The urgency of implementing health policies based on diverse communication channels that cater to the expectations of different subpopulations cannot be deferred.

When planning a public health intervention, it is crucial to undertake evidence-based action. This ensures that adequate and understandable messages are transmitted to the target population, highlighting the significance of our role in public health.

In this context, the World Health Organization (WHO) published a guide in 2023 that tailors health-related services, policies, and communication to enhance specific healthcare topics [113].

“Tailoring health programs” (THPs) were created in 2012 for specific health programs in Europe (vaccination and antimicrobial resistance). However, over the last 10 years, they have spread to several countries with modernized versions. In its 2023 paper, the WHO approved the THP approach for promoting any health program worldwide.

THPs are made up of four phases:

1. Situation analysis: This concerns the health problem that the THP should address and the identification of the target population. Reliable insights about the health problem topic should be analyzed and synthesized, considering the different geographical and sociodemographic patterns. The target population and behavior should be carefully listed.
2. Research: This involves planning the research, including the timing and budget. This phase aims to discover the barriers and drivers of the target population’s inquired behavior.
3. Intervention design: This involves the translation of the work conducted during phase 1 and phase 2 to practical interventions. In particular, the first step should involve selecting and solving the most important barriers of the target group. A detailed project plan should be drawn up and monitored progressively over time.
4. Implementation and evaluation: This includes evaluating the impact of the interventions on the target population in the short and long term. In particular, it should be verified whether the desired change has occurred in the target population. Furthermore, an improvement in health outcomes over the years should be recorded.

**Figure 7:**
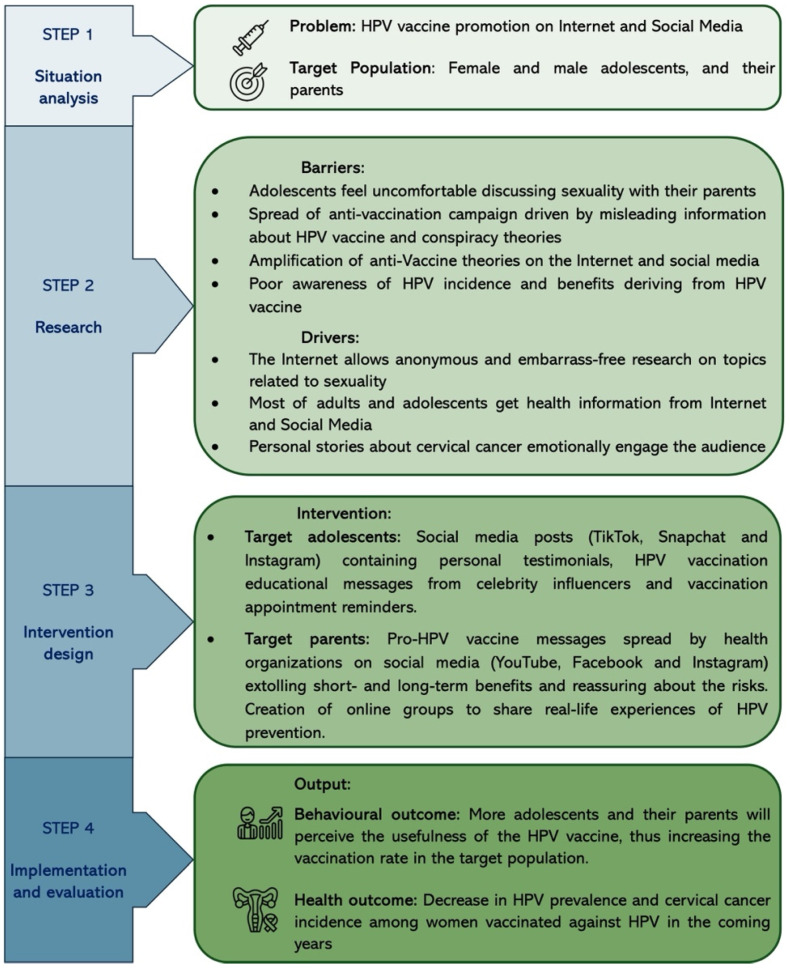
Application of the THP approach in a healthcare program to promote HPV vaccination on the Internet and social media [113]. Icons were obtained from ww.Flaticons.com.

Figure 7 shows an example of the application of the THP approach to promote HPV vaccination on social media.

## 6. Conclusions

The Internet and social media, as compelling information tools in the health field, significantly impact the real world. In the context of HPV vaccination, the type of content can influence the decisions of parents and adolescents about adherence to primary cervical cancer prevention. Health professionals and authorities have a key role to play in the content of online messages. To date, this role is lacking. There is a need to monitor and implement effective strategies to reduce vaccine hesitancy resulting from misleading online messages.

## Supplementary Materials

The following supporting information can be downloaded at: https://www.mdpi.com/article/10.3390/vaccines13050445/s1, Table S1: Detailed summary of selected studies. References [12,18,19,20,22,23,24,25,26,27,28,29,30,31,32,33,34,35,36,37,38,39,40,41,42,43,44,45,46,47,48,49,50,51,52,53,54,55,56,57,58,59,60,61,62,63,64,65,66,67,68,69,70,71,72,73,74,75,76,77,78,79,80,81,82,83,84,85,86,87,88] are cited in the supplementary materials.
