# Supplementary material for: The Role of Internet Information on Anti-HPV Vaccines: A Comprehensive Overview of a Double-Edged Sword
Source: Vaccines (Basel). 2025 Apr 23;13(5):445. doi: 10.3390/vaccines13050445 (PMC12116087; doi:10.3390/vaccines13050445)
Supplement: Supplementary file 1 [file vaccines-13-00445-s001.zip › vaccines-3522908-supplementary.pdf]

Table S1. Detailed summary of selected studies.

Year [ref]	Author	Author's nationality	Study type	Country-data origin	Information topics associated with vaccine hesitancy	Information topics not associated with vaccine hesitancy	Critical issues of Health National or International Agencies on online information
2024 [18]	Kyei GK et al	USA	Review	Unspecified	Side effects	-	-
2024 [22]	Liebermann E et al	USA	Original article	USA	Misleading interpretation of the information source	Social Media type	-
2024 [88]	Boatman et al	USA	Original article	USA	• Conspiracy theories<br>• Side effects<br>• Mistrust of authority<br>• Unnecessary vaccine	-	-
2024 [23]	Lin Z et al	China	Original article	China	-	• Positive personal stories<br>• Careful evaluation of the information veracity	-
2024 [24]	Dang JHD et al	USA	Original article	USA	• Side effects<br>• Mistrust of authority<br>• Unnecessary vaccine	-	-
2024 [25]	Lin Z et al	China	Original article	China	-	• Positive personal stories<br>• Pro-vaccination messages from authoritative sources<br>• Careful evaluation of the information veracity	-
2023 [26]	McKenzie AH et al	USA	Original article	USA	Impact on children's sexual activity	-	-

2023 [27]	Boucher JC et al	Canada	Original article	Unspecified	• Tone of the message (negative, aggressive)<br>• Mistrust of authority	Social Media type	Vaccination forced by Local Health Agencies and WHO
2023 [28]	Coman IA et al	USA	Original article	Unspecified	-	• Social Media type<br>• Anti-stigma Testimonials about HPV infection<br>• Pro-vaccination messages from authoritative sources	• Lack of response to comments or questions from health-related organizations<br>• Lack of personal stories
2023 [29]	Feng Y et al	China	Original article	China	-	• Pro-vaccination messages from authoritative sources<br>• Fear about the consequences of HPV infection (diseases, treatments)	-
2022 [30]	Kim SJ et al	USA	Orginal article	USA	-	• Pro-vaccination messages from authoritative sources	-
2022 [31]	Llavona-Ortiz JY et al	USA	Original article	USA	Individual profile posts	• Pro-vaccination messages from authoritative sources	-
2022 [32]	Leader AE et al	USA	Original article	USA	-	• Positive personal stories	-
2022 [33]	Thompson EL et al	USA	Original article	USA	-	• Careful evaluation of the information veracity	-
2022 [86]	Hu J et al	China	Original article	China	-	• Positive personal stories	-
2022 [34]	Kornides ML et al	USA	Original article	Unspecified	• Side effects<br>• Unnecessary vaccine<br>• Omitting information	-	-

2022 [35]	Oh SH et al	South Korea	Original article	South Korea	-	-	Poor monitoring and reporting online disinformation
2022 [36]	Manganello JA et al	Albany	Original article	USA	-	Social media type	-
2022 [37]	Weinzierl MA et al	USA	Original article	Unspecified	• Conspiracy theories<br>• Unnecessary vaccine	-	-
2022 [38]	Sobzek K et al	Poland	Original article	Unspecified	Tone of the message (negative, aggressive)	-	-
2021 [39]	Zhang J et al	USA	Original article	USA	• Side effects<br>• Mistrust of authority	-	Lack of response to comments or questions from health-related organizations
2021 [40]	Buller DB et al	USA	Original article	USA	-	• Pro-vaccination messages from authoritative sources<br>• Fear about the consequences of HPV infection (diseases, treatments)<br>• Positive personal stories	-
2021 [41]	Sundstrom B et al	USA	Original article	USA	-	• Pro-vaccination messages from authoritatives sources<br>• Discussion/messaging	-
2021 [42]	Chen L et al	China	Original article	Unspecified		Fear about the consequences of HPV infection (diseases, treatments)	-
2021 [43]	van Boetzelaer E et al	Norway	Original article	Norway	-	Instruction/Cultural factors	-

2021 [44]	Jiang S et al	China	Original article	China	-	• Instruction/Cultural factors<br>• Discussion/messaging	-
2021 [45]	Tung WC et al	USA	Original article	China	-	• Social media type<br>• Instruction/cultural factors	-
2021 [46]	Zhou F et al	China	Original article	China	-	-	Poor monitoring and reporting of online disinformation
2021 [47]	Tomaszewski T et al	USA	Original article	Unspecified	Side effects	Positive vaccine effects	-
2021 [48]	Pho AT et al	USA	Original article	USA	-	Social media type	-
2021 [49]	Du J et al	USA	Original article	Unspecified	• Side effects<br>• Conspiracy theories<br>• Omitting information	-	-
2021 [50]	Massey PM et al	USA	Original article	USA	Low socioeconomic status	Careful evaluation of the information veracity	-
2021 [51]	Xiao X e al	China	Original article	Unspecified	Instruction/cultural factors	• Discussing/messaging<br>• Instruction/cultural factors	-
2021 [52]	Argyris YA et al	USA	Original article	USA	Misleading interpretation of information source	-	-
2021 [19]	Wawrzuta D et al	Poland	Review	Unspecified	• Conspiracy theories<br>• Unnecessary vaccine<br>• Side effects	-	-
2021 [53]	Sundstrom B et al	USA	Original article	USA	• Side effects<br>• Personal stories	• Discussion/messaging<br>• Positive personal stories	
2021 [54]	Scherer LD et al	USA	Original article	USA	Instruction/cultural factors	Instruction/cultural factors	-

2021 [55]	Spanos KE et al	USA	Original article	USA	-	-	Poor monitoring and reporting of online disinformation
2021 [56]	Calo WA et al	USA	Original article	USA	Misleading interpretation of the information source	Pro-vaccination messages from authoritative sources	-
2020 [57]	Pan S et al	China	Original article	USA and China	Misleading interpretation of the information source	-	-
2020 [58]	Brandt HM et al	USA	Original article	USA	-	Discussion/messaging	-
2020 [59]	Luisi MLR et al	USA	Original article	Unspecified	• Tone of the message (negative, aggressive)<br>• Individual profile posts	-	-
2020 [60]	Walker KK et al	USA	Original article	USA	• Mistrust of Authority<br>• Unnecessary vaccine<br>• Misleading interpretation of the information source	• Positive personal stories<br>• Pro-vaccination messages from authoritative sources	Poor monitoring and reporting of online disinformation
2020 [61]	Massey PM et al	USA	Original article	Unspecified	• Side effects<br>• Individual profile posts<br>• Conspirancy theories	Positive personal stories	-
2020 [62]	Chodick G et al	Israel	Original article	Israel	Low socioeconomic status	High socioeconomical status	-
2020 [63]	Guidry JPD et al	USA	Original article	Unspecified	• Conspiracy theories<br>• Side effects	Pro-vaccination messages from authoritative sources	-
2020 [64]	Kim SC et al	USA	Original article	USA	-	Humor corrections	-
2020 [65]	Allen JD et al	USA	Original article	USA	Low socioeconomical status	-	-
2020 [66]	Pedersen EA et al	Denmark	Original article	Denmark	-	Positive personal stories	-
2020 [67]	Luisi MLR et al	USA	Original article	Unspecified	Tone of the message (negative, aggressive)	Fear about the consequences of HPV	-

						infection (diseases, treatments)	
2020 [68]	Loft LH et al	Denmark	Original article	Unspecified	-	• Positive personal stories<br>• Discussion/messaging	-
2020 [69]	Chen L et al	USA	Original article	China	Conspiracy theories	Instruction/cultural factors	-
2019 [70]	Dyda A et al	Australia	Original article	Australia	• Conspiracy theories<br>• Tone of the message (negative, aggressive)<br>• Misleading interpretation of the information source	• Discussion/messaging<br>• Pro-vaccination messages from authoritative sources	-
2019 [71]	Buller DB et al	USA	Original article	Unspecified	• Side effects<br>• Mistrust of Authority<br>• Impact on children's sexual activity	Positive personal stories	-
2019 [12]	Ortiz RR et al	USA	Review	Unspecified	• Social media type<br>• Side effects<br>• Conspiracy theories	Pro-vaccination messages from authoritative sources	Poor monitoring and reporting of online disinformation
2019 [72]	Le GM et al	USA	Original article	Unspecified	• Conspiracy theories<br>• Mistrust of Authority	-	-
2018 [87]	Ekram S et al	USA	Original article	Unspecified	• Conspiracy theories<br>• Tone of the message (negative, aggressive)<br>• Side effects	-	-
2018 [73]	Margolis MA et al	USA	Original article	USA	Side effects	-	-
2018 [20]	Perez S et al	Canada	Review	Unspecified	• Impact on children's sexual activity	-	-

					• Misleading interpretation of the information source<br>• Personal stories		
2018 [74]	Mohanty S et al	USA	Original article	USA	-	Pro-vaccination messages from authoritative sources	-
2017 [75]	Kang GJ et al	USA	Original article	USA	• Conspiracy Theories<br>• Mistrust of authority	• Positive vaccine effects<br>• Fear about the consequences of HPV infection (diseases, treatments)	-
2017 [76]	Dunn AG et al	Australia	Original article	USA	• Conspiracy Theories<br>• Side effects	-	-
2017 [77]	Keim-Malpas J et al	USA	Original article	Unspecified	• Mistrust of authority<br>• Side effects<br>• Misleading interpretation of the information source	• Pro-vaccination messages from authoritative sources<br>• Fear about the consequences of HPV infection (diseases, treatments)	-
2016 [78]	Massey PM et al	USA	Original article	Unspecified	• Side effects	• Positive vaccine effects	-
2016 [79]	Surian D et al	Australia	Original article	Unspecified	• Conspiracy theories<br>• Side effects	• Positive vaccine effects	-
2015 [80]	Dunn AG et al	Australia	Original article	Unspecified	• Personal stories<br>• Side effects<br>• Unnecessary vaccine	-	-
2015 [81]	Mahoney LM et al	USA	Original article	Unspecified	• Side effects	• Positive vaccine effects	-
2014 [85]	Pența MA	Romania	Original article	Romania	• Personal stories<br>• Misleading information of the information source	• Positive personal stories	-

						• Pro-vaccination messages from authoritative sources	
2014 [82]	Remschmidt C et al	Germany	Original article	Germany	• Side effects	• Pro-vaccination messages from authoritative sources<br>• Positive vaccine effects	-
2014 [83]	Nakada H et al	Japan	Original article	Japan	• Side effects	-	-
2010 [84]	Keelan J et al	Canada	Original article	Unspecified	• Side effects<br>• Misleading interpretation of the information source	• Positive vaccine effects	-
